# The effect of rivaroxaban on biomarkers in blood and plasma: a review of preclinical and clinical evidence

**DOI:** 10.1007/s11239-023-02776-z

**Published:** 2023-02-06

**Authors:** Sonja Schiffer, Stephan Schwers, Stefan Heitmeier

**Affiliations:** Bayer AG, Pharmaceuticals, R&D, 42113 Wuppertal, Germany

**Keywords:** Biomarkers, Rivaroxaban, Thrombin, Coagulation, Inflammation, Platelets, Endothelial

## Abstract

Rivaroxaban is a direct, oral factor Xa inhibitor that is used for the prevention and treatment of various thromboembolic disorders. Several preclinical and clinical studies have utilized specific molecules as biomarkers to investigate the potential role of rivaroxaban beyond its anticoagulant activity and across a range of biological processes. The aim of this review is to summarize the existing evidence regarding the use of blood-based biomarkers to characterize the effects of rivaroxaban on coagulation and other pathways, including platelet activation, inflammation and endothelial effects. After a literature search using PubMed, almost 100 preclinical and clinical studies were identified that investigated the effects of rivaroxaban using molecular biomarkers. In agreement with the preclinical data, clinical studies reported a trend for reduction in the blood concentrations of D-dimers, thrombin–antithrombin complex and prothrombin fragment 1 + 2 following treatment with rivaroxaban in both healthy individuals and those with various chronic conditions. Preclinical and also some clinical studies have also reported a potential impact of rivaroxaban on the concentrations of platelet activation biomarkers (von Willebrand factor, P-selectin and thrombomodulin), endothelial activation biomarkers (matrix metalloproteinase-9, intercellular adhesion molecule-1 and vascular cell adhesion molecule-1) and inflammation biomarkers (interleukin-6, tumor necrosis factor-α and monocyte chemoattractant protein-1). Based on the results of biomarker studies, molecular biomarkers can be used in addition to traditional coagulation assays to increase the understanding of the anticoagulation effects of rivaroxaban. Moreover, there is preliminary evidence to suggest that rivaroxaban may have an impact on the biological pathways of platelet activation, endothelial activation and inflammation; however, owing to paucity of clinical data to investigate the trends reported in preclinical studies, further investigation is required to clarify these observations.

## Highlights

- Rivaroxaban, a direct, oral factor Xa inhibitor used for the prevention and treatment of various thromboembolic disorders potentially plays a role beyond its anticoagulant activity and across a range of biological processes.
- A literature search with the main focus on Medline via PubMed was done to summarize the existing evidence regarding the use of blood-based biomarkers to characterize the effects of rivaroxaban on coagulation and other pathways, including platelet activation, inflammation and endothelial effects in clinical and preclinical studies.
- In preclinical and clinical studies not only a trend for reduction in the blood concentrations of some relevant coagulation biomarkers (D-dimers, thrombin–antithrombin complex and prothrombin fragment 1+2) following treatment with rivaroxaban was reported, but also for a potential impact of rivaroxaban on the concentrations of platelet activation biomarkers (von Willebrand factor, P-selectin and thrombomodulin), endothelial activation biomarkers (matrix metalloproteinase-9, intercellular adhesion molecule-1 and vascular cell adhesion molecule-1) and inflammation biomarkers (interleukin-6, tumor necrosis factor-α and monocyte chemoattractant protein-1).
- Clinical impact of those findings is work in progress.

## Background

The coagulation cascade consists of a sequence of consecutive protease activations steps ultimately leading to the generation of fibrin and contributing to platelet activation, the two main processes in blood clotting. A well-functioning coagulation cascade is central to maintaining hemostasis [1]. Conditions which can lead to prothrombotic states, including diseases like atrial fibrillation (AF) and acute coronary syndrome (ACS) or procedures resulting in immobility, such as hip or knee surgery [2–4] may cause hypercoagulability increasing the risk of development of life-threatening thrombosis [1, 5].

Rivaroxaban is a direct, oral anticoagulant that is used for the prevention and treatment of various thromboembolic disorders. The mode of action involves the reversible inhibition of activated factor Xa (FXa), a key component of the blood coagulation pathway [6, 7]. Licensed indications for rivaroxaban include: Treatment of pulmonary embolism (PE) or deep vein thrombosis (DVT); prevention of recurrence of PE or DVT; prevention of systemic embolism or stroke in patients with nonvalvular AF; prophylaxis of VTE in patients undergoing hip or knee replacement surgery and prevention of atherothrombotic events after ACS and in CAD/symptomatic PAD [2, 8–10].

Coagulation factors have also been implicated in other biological processes, such as tissue repair, platelet activation and inflammation [11, 12]. As such, it has been hypothesized that rivaroxaban could have additional impacts on a range of those biological processes. One approach that can be used to investigate the potential effects of rivaroxaban on other biological pathways is through the measurement of biomarkers, molecules known to provide insights into the status of these processes. Studies of biomarkers can provide information regarding ongoing biological changes and can improve understanding of the mode of action of drugs. Disease-related and drug-related pharmacodynamic biomarkers have been used to help understand and predict patients’ characteristics and risk, optimize patients’ selection, drug dosing and improve decision-making throughout the drug development process [13]. Furthermore, the capabilities of assays in the preclinical setting have allowed for the exploratory investigation of potential biomarkers to understand a variety of processes which could potentially be translated into the clinic.

The aim of this literature review is to summarize the evidence for the impact of rivaroxaban on various pathways; specifically, we describe studies exploring how molecular biomarkers may be used to further characterize the effects of rivaroxaban on coagulation and on other biological processes.

## Methods

To inform the discussions in this review, searches were conducted in Medline mainly via PubMed to identify literature reporting preclinical and clinical studies of rivaroxaban and biomarkers of coagulation, platelet activation, inflammation, endothelial changes and other biological processes. The following search strings were used.

1. (rivaroxaban) AND (biomarker) [All Fields] (January 2022)
2. (rivaroxaban[Title/Abstract]) AND ((oxidation[Title] OR oxidant[Title] OR platelet[Title] OR endothelial[Title]) OR (inflammation[Title] OR inflammatory[Title] OR cytokine[Title] OR leukocyte[Title]) OR (coagulant[Title] OR coagulation[Title] OR d dimer[Title] OR prothrombin[Title] OR viper venom[Title])) (January 2022)

The references retrieved from these searches were screened and a total of 97 studies were identified. The search results were confirmed by similar search terms in other databases including Google Scholar. In addition to these searches, reference lists from published literature reviews were cross-checked to identify studies that discussed disease biomarkers for various indications of rivaroxaban (January 2022).

### The coagulation cascade

The coagulation cascade is a complex sequence of events. Blood clotting is initiated when either subendothelial tissue factor (TF) gets into contact with blood after vessel wall injury, binds activated factor VIIa (FVIIa) and activates factor IX and factor X (FX) [7] or in the cause of contact activation, i.e. FXII is activated on negatively charged surfaces, leading to factor XI- and FIX activation. As a result of such signaling, FX is activated to FXa and catalyzes thrombin generation by acting as part of the prothrombinase complex; thrombin is subsequently responsible for the conversion of fibrinogen to fibrin, a central process in blood clot formation [14]. Rivaroxaban has been shown to inhibit FXa, irrespective of whether it is free or bound in the prothrombinase complex, resulting in reduced thrombin generation, thereby prolonging blood clotting times [14–16].

### Global/functional assays of rivaroxaban activity

Various assays can be used to assess the functionality of the coagulation cascade. While most of these assays use the development of a fibrin clot as common endpoint, it is the trigger and thereby the starting point, which varies. Some assays like the prothrombin time (used to either measure the extrinsically triggered coagulation or monitor the impact of Vitamin K antagonists), or activated partial thromboplastin time (used to either measure the intrinsic pathway or the impact of heparin therapy) are among the most frequently performed tests in the clinical laboratory and are available even through handheld self-testing devices. Other methods are rarely used—only to focus on specific questions. While most of those assays are usually performed from anticoagulated (mostly citrated) plasma, ROTEM (rotational thromboelastometry) and TEG (thromboelastography) are whole blood tests, which are most often used in critical care setting. The thrombin generation assay, usually only available in specialized labs, allows to determine the formation of thrombin as key enzyme of the coagulation over time, thereby providing better insights into kinetic aspects of coagulation.

**Fig. 1:**
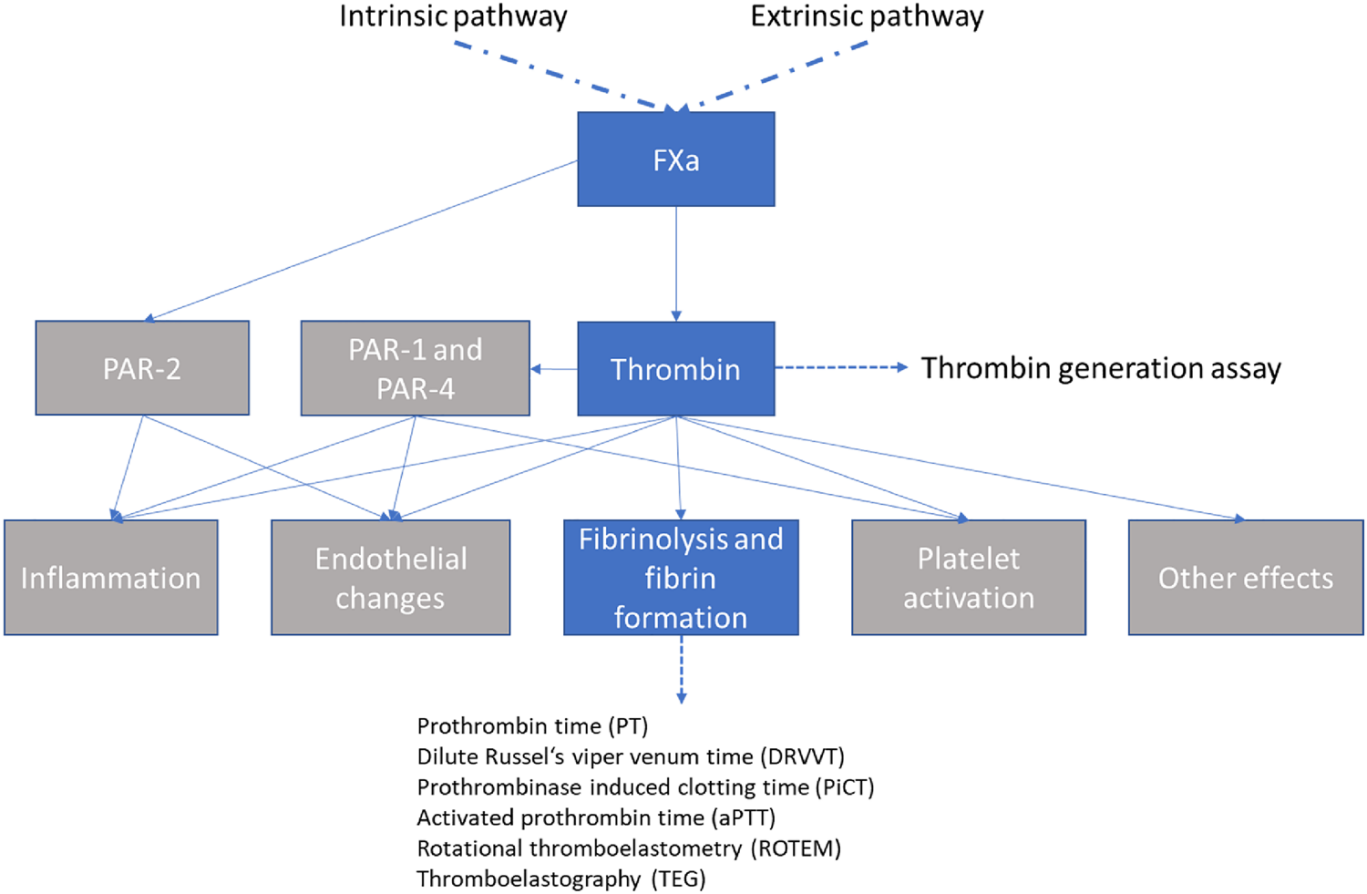
The coagulation cascade and associated functional assays [1, 33, 43]

Figure 1 provides an overview of these types of methods and the stages of the coagulation pathways that they measure. From the earliest experiments with rivaroxaban on it has been known that the compound prolongs the clotting times dose-dependently. Table 1 lists examples of studies that have reported the anticoagulation effects of rivaroxaban, specifically through the use of functional coagulation assays [10, 17–42].

**Table 1:** Selected studies that have investigated the anticoagulation effects of rivaroxaban with global/functional coagulation assays

Study ID	Disease setting	Assay						
		TG	PT	aPTT	ETP	RVVT	PiCT	ROTEM/ TEG
*Clinical studies*								
Arachchillage et al. 2015 [17]	VTE	✔	✔	–	✔	–	–	–
Ebner et al. 2017 [18]	Patients treated with DOAC in clinical practice	–	✔	✔	–	–	–	–
Gheorghiade et al. 2011 [19]	Heart failure	–	✔	–	–	–	✔	–
Graff et al. 2007 [20]	Healthy volunteers	–	✔	✔	✔	–	✔	–
Green et al. 2010 [21]	Prophylaxis after hip/knee surgery	✔	✔	✔	✔	–	–	–
Hagii et al. 2016 [22]	Acute cardioembolic stroke	–	✔	–	–	–	–	–
Helin et al. 2017 [23]	Total hip arthroplasty	✔	✔	✔	✔	✔	–	–
Hitaka et al. 2016 [24]	Nonvalvular AF	–	✔	✔	–	–	–	–
J-ROCKET (Chan et al. 2012) [25]	Nonvalvular AF	–	✔	–	–	–	✔	–
Katoh et al. 2017 [26]	Nonvalvular AF	–	✔	✔	–	–	–	–
Kitagawa et al. 2017 [27]	Nonvalvular AF	–	✔	✔	–	–	–	–
Mani et al. 2011 [28]	Total hip or knee arthroplasty	–	✔	✔	–	–	–	–
Molenaar et al. 2012 [29]	Healthy volunteers	✔	✔	✔	✔	–	–	–
Nakano et al. 2015 [30]	Nonvalvular AF		✔	–	–	–	–	–
Oswald et al. 2015 [31]	Major orthopedic surgery	✔	–	–	✔	–	–	✔
Platton et al. 2017 [32]	Patients receiving rivaroxaban	–	✔	✔	–	–	–	–
Pratt et al. 2018 [33]	Patients receiving DOACs who presented at emergency rooms	–	✔	✔	–	✔	–	–
Steppich et al. 2017 [34]	Nonvalvular AF	✔	–	–	–	–	–	–
Suzuki et al. 2018 [35]	Nonvalvular AF	–	✔	–	–	–	–	–
Tajiri et al. 2015 [36]	Nonvalvular AF	–	✔	✔	–	–	–	–
Wan et al. 2016 [37]	Healthy volunteers	✔			✔	–	–	–
X-PLORER (Vranckx et al. 2015) [38]	CAD	–	✔	✔	✔	–	–	–
Hirota et al. 2020 [44]	Nonvalvular AF	–	✔	–	–	–	–	
Borst et al. 2018 [10]	Non-ST elevation myocardial infarction	✔	–	–	–	–	–	–
*Preclinical studies*								
Douxfils et al. 2012 [39]	In vitro – human plasma	✔	✔	✔	–	–	✔	–
Parry et al. 2011 [40]	Rat model of arterial thrombosis	–	✔	✔	–	✔	–	–
Rosenkranz et al. 2011 [41]	In vitro – vascular smooth muscle cells	✔	–	–	–	–	–	–
Sparkenbaugh et al. 2014 [42]	Mouse model of sickle cell disease	–	✔	✔	–	–	–	–
Perzborn et al. 2005 [16]	Rat venous stasis model, Arteriovenous shunt model in rats and rabbits	–	✔	✔	–	–	–	–
Biemond et al. 2007 [45]	Rabbit venous stasis model, rabbit jugular vein thrombosis model	–	✔	–	–	–	–	–
Zhou et al. 2013 [46]	Murine model of intracerebral hemorrhage	–	✔	–	–	–	–	–

*AF* atrial fibrillation; *aPTT* activated partial thromboplastin time; *CAD* coronary artery disease; *DOAC* direct oral anticoagulant; *ETP* endogenous thrombin potential; *ID* identifier; *PiCT* prothrombinase-induced clotting time; *PT* prothrombin time; *ROTEM* rotational thromboelastometry; *RVVT* Russell’s viper venom time; *TEG* thromboelastography; *TG* thrombin generation; *VTE* venous thromboembolism

### Molecular biomarkers of rivaroxaban activity

**Fig. 2:**
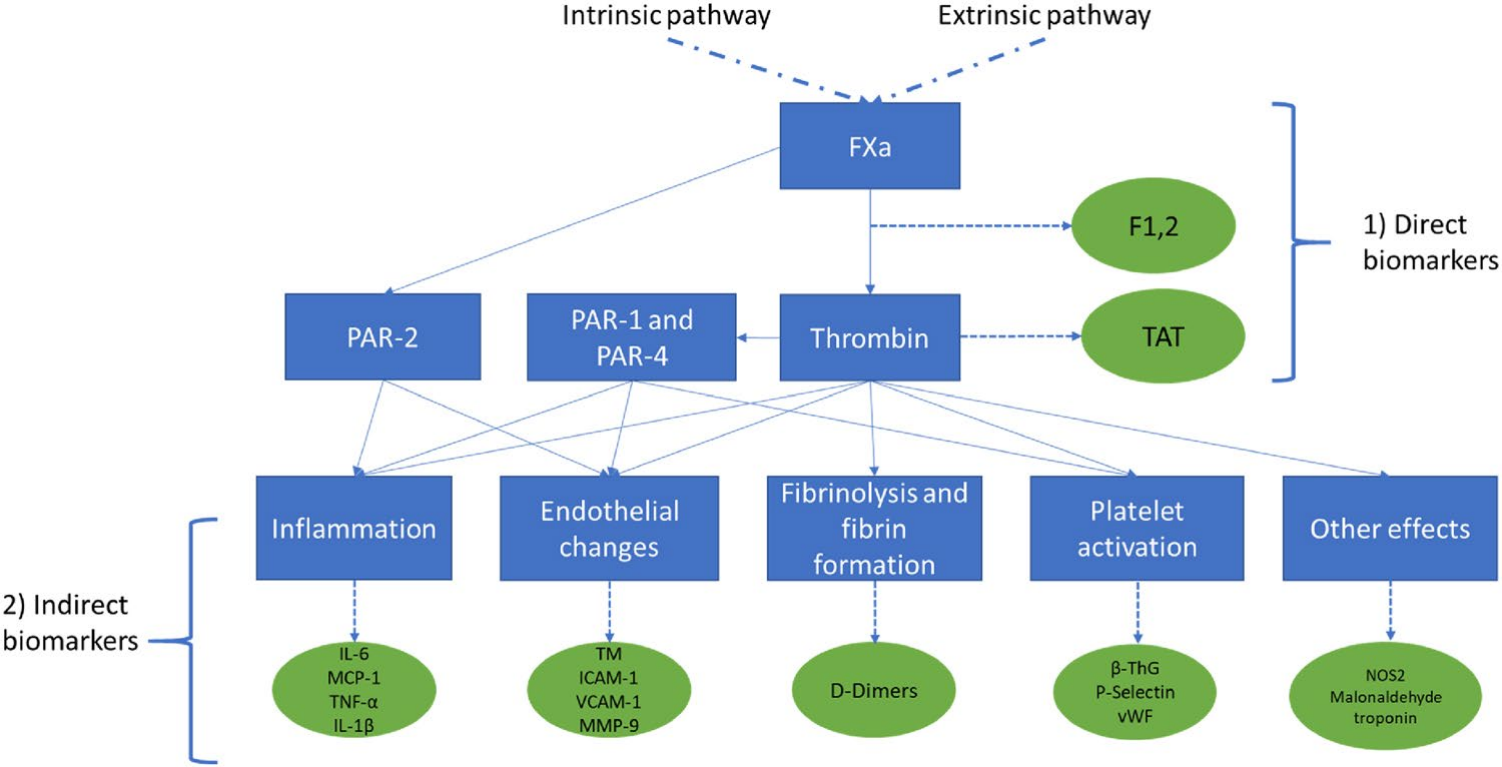
The coagulation cascade and the associated molecular biomarkers

In addition to the use of global/functional assays to assess the coagulation status, the impact of rivaroxaban has been investigated by measuring concentrations of specific biomarkers, see Fig. 2. Molecular biomarkers may provide supplementary information regarding the effects of rivaroxaban because they allow for a snapshot of the concentrations of coagulation products. Examples of the investigated molecular biomarkers are depicted in Fig. 2 and include different types:

- Direct markers: Biomarkers, that are directly affected by the mode-of-action of rivaroxaban, i.e. as a proximate result of FXa inhibition and subsequent reduction of thrombin formation.
- Indirect markers: Biomarkers that indicate biological processes downstream of FXa and thrombin, either within the coagulation cascade (e.g. D-Dimer) or outside (e.g. inflammatory markers).

Direct markers are directly affected by the mode-of-action of rivaroxaban, i.e. as a proximate result of FXa inhibition and subsequent reduction of thrombin formation. Indirect markers indicate biological processes downstream of FXa and thrombin, either within the coagulation cascade (e.g. D-Dimer) or outside (e.g. inflammatory markers).

F1 + 2, prothrombin fragment 1 + 2; FXa, activated factor X; ICAM-1, intercellular adhesion molecule-1; IL-1β, interleukin 1β; IL-6, interleukin-6; MCP-1, monocyte chemoattractant protein-1; MMP-9, matrix metalloproteinase-9; NOS2; nitric oxide synthase isotope 2; PAR, protease-activated receptor; TAT, thrombin–antithrombin complex; β-ThG, thromboglobulin; TM, thrombomodulin; TNF-α, tumor necrosis factor-α; VCAM-1, vascular cell adhesion molecule-1; vWF, von Willebrand factor.

#### Direct biomarkers

The biomarkers Russel’s viper venum (RVV) FX test, prothrombin fragment 1 + 2 (F1 + 2) and thrombin–antithrombin (TAT) complex are directly derived from substrates of the activated coagulation pathway by FXa. Studies have investigated the effects of rivaroxaban on the concentrations of these molecular biomarkers.

##### Russell’s viper venom (RVV) FX test.

Russell's Viper Venom is an enzyme extracted from snake venom (*Daboia russelii)* and activates Factor X directly. In the presence of Factor V, Prothrombin, Calcium and Phospholipid a fibrin clot is formed. Within the assay, Factor Xa specifically cleaves a substrate which can be optically measured. This reaction can be used to directly assess the effect of Rivaroxaban on FXa activity in plasma. Hence, it was shown clinically that Russell viper venom reagents correlate with rivaroxaban concentration and that they can reflect the inhibition of FXa in pre-clinical models [16, 47].

##### Prothrombin fragment 1 + 2 (F1 + 2).

F1 + 2 is a peptide cleaved from the amino-terminal end of prothrombin by FXa during activation to thrombin. As such, the concentration of F1 + 2 in the plasma reflects in vivo thrombin generation [48].

There is some clinical evidence of F1 + 2 as a useful molecular biomarker to assess the effect of rivaroxaban. Several clinical studies have reported reductions from baseline in the plasma concentration of F1 + 2 in rivaroxaban-treated patients with heart failure (HF) [19], AF [27, 49] or acute cardioembolic stroke [22], as well as in healthy individuals [50, 51] and in patients after hip or knee replacement surgery [21]. For example, in a study conducted by Gheorghiade et al*.* [19], in patients with HF who were treated with rivaroxaban, the mean concentration of F1 + 2 decreased by 2.7 ng/mL over 7 days compared with an increase of 11.6 ng/mL in patients who received placebo (*p* = 0.0009 for the difference between groups) [19]. Similarly, a study in patients with AF reported lower mean concentrations of F1 + 2 in circulating plasma in patients treated with rivaroxaban (103.5 pmol/L) than in patients not treated with anticoagulants (162.5 pmol/L) [52]. However, not all studies have reported this trend: the study of Miyazawa et al. [53] in patients with nonvalvular AF and left atrial/left atrial appendage (LA/LAA) thrombus, who received rivaroxaban 20 mg once daily, found no significant baseline-adjusted differences in the mean concentrations of F1 + 2 [53].

Studies have also compared the respective effects of warfarin and rivaroxaban treatment on plasma levels of F1 + 2 in patients with AF. These investigations reported lower plasma levels of F1 + 2 in warfarin-treated patients than in rivaroxaban-treated patients. One hypothesis for this finding is that the pharmacokinetic profiles of the two compounds might lead to differences in the interaction with the kinetics of F1 + 2, e.g. mean half-life of Rivaroxaban is 5–13 h and of Warfarin 40 h. [24, 36, 54]. Similarly, a study in patients with cardioembolic stroke receiving rivaroxaban or warfarin for secondary stroke prevention did not find any rivaroxaban-treated patients with levels of F1 + 2 below the normal range, irrespective of dosage. However, many patients receiving warfarin had levels below the normal range. As such, the authors proposed that normal thrombin generation may be preserved even when rivaroxaban treatment is at its peak level; this could partly explain the observed, favorable outcomes in rivaroxaban-treated patients with intracranial hemorrhage [22].

##### Thrombin–antithrombin III complex.

Levels of thrombin are regulated by a variety of physiological inhibitors; the main inhibitor of thrombin is antithrombin III (ATIII) [55, 56]. Antithrombin and thrombin form equimolar thrombin-antithrombin III complexes (TAT) which lead to the inactivation of thrombin in blood. The concentrations of TAT in the blood have been shown to reflect the formation of thrombin [56, 57].Preclinical evidence in animal models generally supports the use of TAT complex concentrations as a biomarker of the anticoagulation effects of rivaroxaban. In some rat and mouse models of hypercoagulation, plasma concentrations of TAT were lower in rivaroxaban-treated animals than in controls [40, 42, 58]. For example, in a rat model of brain ischemia/reperfusion injury reported by Dittmeier et al. [58], mean concentrations of TAT in the brain were significantly lower 1 day after stroke in rats pretreated with rivaroxaban than in vehicle-treated controls (3057.0 pg/mL vs 5048.0 pg/mL; *p* < 0.05) [58]. Conversely, in a study of apolipoprotein E-deficient mice, Hara et al. [59] reported no difference in plasma concentrations of TAT between rivaroxaban-treated mice and controls [59]. The impact of rivaroxaban on the TAT concentrations in these different animal experiments may depend on the dose of rivaroxaban, the animal species used in the study and importantly the different pathologies involved. For instance, thrombosis studies with high TAT levels in the control group share higher chances to yield significant TAT level reductions with rivaroxaban, whereas experiments yielding low TAT levels in the control group, like atherosclerotic experiments without thrombotic event, may result in low TAT reduction levels with rivaroxaban.

As anticipated by the preclinical evidence, clinical studies have also reported a trend for reduction in TAT complex levels following treatment with rivaroxaban. Reductions in the plasma concentrations of TAT complex from baseline after rivaroxaban treatment have been observed in patients with nonvalvular AF [53, 54] in healthy individuals [50] and in patients after hip or knee replacement surgery [21]. In the study of healthy individuals conducted by Weisshaar et al. [50], single doses of rivaroxaban (combined with ticagrelor and acetylsalicylic acid) significantly reduced concentrations of TAT complex in shed blood at 3 h after treatment (median, 127 µg/L at 3 h vs 630 µg/L at baseline; *p* < 0.001) [50]. Similarly, in a study of rivaroxaban-treated patients undergoing percutaneous coronary intervention (PCI), concentrations of TAT complex were suppressed after the PCI [38].

##### Protease-activated receptors-1, -2 and -4.

Initiation of the coagulation cascade results in the activation of platelets and upregulation of adhesion molecules and pro-inflammatory pathways in blood, which in turn accelerates the coagulation processes. Key to this signaling is the cleavage of peptides from protease-activated receptors by FXa and thrombin liberating the tethered ligands, which activate the respective receptor. Thrombin activates by this means protease-activated receptors-1 and -4 (PAR-1 and PAR-4) and FXa PAR-2 and (weakly) PAR-1 (Fig. 2) [60, 61]. In addition, PAR-2 seems to be activatable by the TF-FVIIa-FXa complex. [62, 63]. Because the receptor activation leads to subsequent internalization, this process cannot be measured easily on the cell surface but might be monitored at later stages of the signaling cascade. The activation of PAR-1 and -4 on platelets leads to their activation, while activation of PAR-1 and -2 receptors on endothelial cells and various other cell types results in proinflammatory signaling through several pathways, which trigger among others the generation of pro-inflammatory molecules.

Preclinical studies have reported some associations between rivaroxaban treatment and a reduction in the concentrations of PARs. For example, in a mouse model of myocardial reperfusion injury, compared with controls, mice treated with rivaroxaban had significantly reduced levels of mRNA for PAR-2 in the left ventricle. Chung et al. showed in an atrial fibrosis model with isoproterenol-treated rats that rivaroxaban decreases collagen production and migratory capability of atrial fibroblasts by increasing NO production and decreasing Ca2 + entry through inhibition of PAR signaling [64]. In vitro studies showed that DOACs, including rivaroxaban, seem to limit the alteration of the monolayer of endothelial cells of the blood brain barrier mediated by the thrombin/PAR-1 pathway [65]. Furthermore it was suggested, that rivaroxaban-mediated inhibition of PAR-1 has a positive impact on atherothrombotic events [66].

#### Indirect biomarkers

Indirect biomarkers can provide insights into biological pathways that are also affected by various players downstream in the coagulation cascade, and thereby provide a window into other processes that may be affected by rivaroxaban. Examples of such molecular markers are depicted in Fig. 2. The following sections summarize the published evidence for the impact of rivaroxaban on molecular biomarkers of fibrin formation and coagulation, platelet activation, inflammation and endothelial changes.

##### D-dimers

D-dimers derive from the cleavage of cross-linked, insoluble fibrin molecules during endovascular thrombosis, one of the last stages in the coagulation cascade. Serum/plasma D-dimer levels have been shown to correlate with extent of thrombolytic activity in the body and the amount of thrombotic deposits [67, 68].

Many clinical studies have demonstrated a reduction in the concentrations of D-dimers in the blood following treatment with rivaroxaban. Reductions in plasma concentrations of D-dimer from baseline after rivaroxaban treatment have been reported in patients with AF [26, 27, 53], ACS [69] or acute cardioembolic stroke [22], as well as in healthy individuals [70]. For example, in a sub-study of patients with ACS from the ATLAS ACS-TIMI 46 trial, reductions from baseline in median D-dimer levels 180 days after treatment initiation were significantly greater (*p* < 0.001) in the rivaroxaban-treated group (− 0.14 µg/mL) than with placebo (− 0.06 µg/mL) [69]. Similarly, in the X-TRA biomarker sub-study of patients with LA/LAA thrombus and AF, patients treated with rivaroxaban had a reduction in plasma concentrations of D-dimer from baseline to the end of treatment (− 41.5%; *p* < 0.001) [53]. In the X-VeRT substudy which evaluated the effects of treatment with rivaroxaban or VKA on levels of different biomarkers of coagulation and inflammation in nonvalvular AF patients scheduled for cardioversion, D-Dimer levels were also decreased by 32.3% (compared to VKA with 37.6%) [54]. In addition, a clinical study conducted by Spyropoulos et al. [71] reported D-dimer levels remaining consistently below the normal cut-off in rivaroxaban-treated patients with venous thromboembolism, while high D-Dimer levels could support the identification of elevated VTE risks in medically ill patients [71]. Also, as shown in the COMMANDER HF trial, D-dimer aided to predict stroke risk and rivaroxaban benefit in a HF patient population [72].

##### Platelet activation

Platelet activation can lead to adhesion and aggregation through platelet receptors. The activation of platelets is a key event during blood clotting and is highly interlinked with the coagulation cascade. During clotting, thrombin can induce activation of PAR-1 and PAR-4, leading to downstream signaling events such as granule secretion, and studies suggest that glycoprotein Ib-IX receptor complex signaling cooperates with PAR signaling to promote platelet activation in response to low thrombin concentrations [73]. Platelets release the contents of granules, including β-thromboglobulin, P-selectin and von Willebrand factor (vWF) [34]. It has been hypothesized that rivaroxaban may affect the platelet activation process, and concentrations of β-thromboglobulin, thrombospondin, vWF and P-selectin have been used as potential biomarkers to address this hypothesis [74, 75].

In an in vitro study in which blood was spiked with rivaroxaban, there was a reduction in P-selectin surface expression, indicating a modest attenuation of thrombin-induced or TF-induced activation of platelets. An additional finding in this study was that the addition of rivaroxaban to blood before adenosine diphosphate (ADP)-induced activation led to limited but consistent attenuation of activation. The authors concluded that these results merit further investigation but suggest that rivaroxaban could interfere directly with ADP-induced platelet activation [76].

Building on the evidence reported in preclinical studies, similar trends for platelet activation biomarkers have been reported in the clinical setting. For example, in a proteomic analysis of rivaroxaban-treated patients with nonvalvular AF, there were significant decreases (*p* = 0.0246) in circulating P-selectin from day 1 to day 24 of treatment [25]. Furthermore, Weisshaar et al*.* [50] reported a trial in healthy individuals randomized to treatment with rivaroxaban. In these participants, concentrations of β-thromboglobulin in shed blood were significantly decreased compared to pre-dose concentrations (1534 IU/mL at baseline vs. 987 IU/mL at 3 h after the dose; *p* ≤ 0.001). Additionally, an analysis of rivaroxaban-treated patients with AF and LA/LAA thrombi reported a significant baseline-adjusted decrease in mean serum concentration of vWF from baseline to end of treatment (− 32%; *p* < 0.001) [53]. Also, Ordi-Ros et al. reported a decrease in the platelet activation biomarker over time (3 years) after treatment of patients with thrombotic Antiphospolipid Syndrome (APS) with Rivaroxaban, whereby vWF levels decrease only slightly. No significant differences were seen here compared to Warfarin treatment [77].

While a trend for reduction in the plasma levels of these platelet activation biomarkers has been reported across various studies, other clinical investigations have failed to find significant changes following rivaroxaban treatment [34, 70, 78]. Specifically, Steppich et al*.* [34] reported a clinical study of patients with nonvalvular AF and found no significant reductions from baseline in the plasma levels of β-thromboglobulin, thrombospondin, vWF or P-selectin after treatment with rivaroxaban [34]. Similarly, in another clinical study of 10 rivaroxaban-treated (and 17 dabigatran-treated) patients with nonvalvular AF, Zemer-Wassercug et al*.* [78] reported no significant differences between baseline and post-rivaroxaban treatment platelet reactivity as measured by the proportion of P-selectin expressed on the platelet membrane [78].

##### Inflammatory activity

Inflammatory processes are closely linked with the blood coagulation cascade. For example, thrombin can interact with PAR-1 on endothelial cells, fibroblasts and monocytes, causing a downstream signaling cascade that triggers production of pro-inflammatory molecules, including monocyte chemoattractant protein-1 (MCP-1), tumor necrosis factor-α (TNF-α), interleukin-1β (IL-1β) and interleukin-6 (IL-6) [79–82]. In addition, PAR-2, expressed by vascular endothelial cells, was found to be involved in thrombin-independent, pro-inflammatory signaling, through interaction with TF and FVIIa [62, 63]. Several studies have investigated pro-inflammatory molecules as potential biomarkers of inflammatory changes following rivaroxaban treatment.

Tissue explant experiments have reported reductions in the levels of mRNA expression relating to inflammatory molecules in rivaroxaban-exposed samples compared with controls, including IL-6 [41, 83, 84], MCP-1 [59, 83, 85, 86], IL-1β [59, 83] and TNF-α [41, 59], and in protein concentrations of IL-6 [87]. However, not all results have shown this trend and an investigation of the cytokines released from monocytes during the process of thrombin generation found that rivaroxaban had no significant influence on IL-6 or TNF-α secretion [88].

In addition to tissue explant studies, investigations using inflammatory animal models have reported reductions in the levels of mRNA expression and plasma concentrations of various molecules in rivaroxaban-treated animals compared with controls, including IL-6 [89–92], MCP-1 [89], IL-1β [58] and TNF-α [58, 59, 89]. Terry et al*.* [93] investigated the effects of rivaroxaban treatment on pro-inflammatory molecule levels in a mouse model of catheter thrombosis. This study reported lower concentrations of MCP-1 protein in rivaroxaban-treated mice than in controls, but plasma levels of IL-6 and TNF-α did not significantly differ between groups [93].

The evidence of anti-inflammatory effects of rivaroxaban in the clinical setting is limited; however, some clinical investigations have provided hints of an association between rivaroxaban treatment and inflammatory processes. For example, in the X-TRA study, elevated levels of high sensitivity IL-6 (hsIL-6) at baseline were significantly associated with thrombus reduction or resolution in rivaroxaban-treated patients with AF (odds ratio, 4.909; *p* = 0.021). However, in this study, rivaroxaban treatment did not lead to significant changes in concentrations of hsIL-6 between baseline and end of rivaroxaban treatment even though the patients in general benefited from rivaroxaban treatment [53]. In the X-VeRT study, reductions of 12.5% and 9.2% for hs-CRP and hs-IL-6 respectively were observed [54]. Similarly, in another study of patients with AF, no significant changes from baseline in blood levels of TNF-α or IL-6 were reported after 6 months of rivaroxaban treatment [26].

##### Endothelial changes

In addition to their role in coagulation, thrombin and FXa elicit multiple effects on endothelial cells, including the modulation of the expression of genes encoding proteins that play a role in adhesion and inflammation either directly by PAR receptor signaling on the endothelial surface or indirectly via PAR-initiated platelet activation and subsequent adhesion once endothelium is damaged and aggregation during blood clot formation [94].

To investigate how rivaroxaban-induced inhibition of FXa and thrombin generation could impact endothelial cells, in addition to the levels of excreted inflammatory biomarkers, biomarkers of endothelial surface activation have been investigated. Cell surface molecules that have been studied include: thrombomodulin, intercellular adhesion molecule-1 (ICAM-1), vascular cell adhesion molecule-1 (VCAM-1) and other extracellular markers such as matrix metalloproteinase (MMP)-9. Thrombomodulin is a protein expressed on endothelial cell surfaces which can bind thrombin, subsequently activate protein C, and inhibit the process of coagulation [12]. ICAM-1 and VCAM-1 are surface adhesion molecules expressed on endothelial cells [85] and MMP-9 is a zinc-dependent enzyme involved in degradation of the extracellular matrix during biological processes. Raised concentrations of MMP-9 protein have been associated with various disease states, including cardiovascular conditions [95, 96].

In in vitro studies the downregulation of ICAM-1 and VCAM-1 mRNA expression following administration of rivaroxaban compared with untreated controls has been reported [59, 83, 85]. In agreement to these data, animal studies have shown reductions in the expression level of ICAM-1 mRNA in the rivaroxaban group compared to control animals [58]. Similarly, preclinical studies have suggested that MMP-9 could be a potential biomarker of the inhibition of endothelial activation by rivaroxaban. In animal models, the expression of MMP-9 was reduced in rivaroxaban-treated animals compared with controls [59, 93]. In addition, in a study conducted by Monux et al. (2017), in vitro incubation of rivaroxaban with samples of human abdominal aortic aneurysm sites with intraluminal thrombus resulted in significantly reduced MMP-9 expression to levels similar to those found in control aortas [87]. However, in contrast, Rosenkranz et al*.* [87] reported no significant effect on MMP-2 and MMP-9 levels in clot-stimulated, vascular smooth muscle cells treated with rivaroxaban [41].

In line with the known antithrombotic role of thrombomodulin, a clinical study in 23 patients with nonvalvular AF reported a significant increase in mean concentrations of plasma thrombomodulin after 6 months of treatment with rivaroxaban (2.9 fibrinolytic units (FU)/mL to 3.2 FU/mL; *p* = 0.003) [26]. In a proteomic analysis of the ROCKET-AF trial, a randomized study of patients with nonvalvular AF receiving rivaroxaban or warfarin treatment, investigators reported upregulation of soluble thrombomodulin during treatment with rivaroxaban compared with treatment with warfarin [25]. This study also reported a significant reduction (*p* = 0.0338) in MMP-9 protein concentrations from baseline to week 24 of rivaroxaban treatment. There was additionally a trend toward a greater decrease in MMP-9 levels in rivaroxaban-treated patients than in warfarin-treated patients [25]. Although these trials provide promising data for a relationship between rivaroxaban treatment and molecular biomarkers of endothelial changes, further clinical studies will be required to confirm these findings.

In an in vitro experiment for diabetic endothelial senescence, HUVECs were cultured with/without rivaroxaban under high glucose (HG). Senescence- associated-β-galactosidase (SA-β-gal), p53, p21, and p16INK4a were increased by HG via PAR receptors and restored by rivaroxaban, which restored telomerase activity and preserved telomere length, as well, suppressed O2–, p22phox, and ICAM1 and restored NOx and eNOS. In dyslipidemic diabetic mice, plasma glucose, total cholesterol, and triglycerides were increased for 4 weeks but were not changed by rivaroxaban. However, rivaroxaban decreased SA-β-gal and telomerase and preserved telomere length in the aortic endothelium. Rivaroxaban activated eNOS, inhibited p22phox, increased plasma NOx, and decreased O2–. Thus, rivaroxaban prevented replicative senescence in HUVECs and aortic endothelial cells, restored endothelial function and prevented the progression of atherosclerosis [97]. A clinical study with type 2 diabetes mellitus and subclinical inflammation showed that rivaroxaban compared to Aspirin could improve endothelial function based different measures such as post-ischaemic forearm blood flow during reactive hyperaemia, skin blood flow, sP-Selectin or platelet-derived microparticles which stimulate endothelial repair [98].

### Disease and organ biomarkers

For many cardiovascular diseases, including those that are indications for rivaroxaban, studies have suggested that disease states are associated with the in vivo levels of molecular biomarkers of coagulation and inflammation. Therefore, many of the molecular biomarkers discussed above could be important as biomarkers for disease severity and progression. Table 2 summarizes some examples of studies that have reported associations between such molecular biomarkers of coagulation and inflammation in patients with cardiovascular disease [19, 99–108].

**Table 2:** Evidence for changes to molecular biomarkers in various disease states

Disease state	Associated biomarkers	Observations reported in the literature
AF	CRP, IL-6, TNF-α, MCP-1, IL-1β	Higher levels of IL-6 have been observed in patients with AF compared with controls (*p* = 0.034) [99] Raised levels of circulating IL-6, CRP and TNF- α were associated with increased AF risk in the general population [100] In a case–control study, serum concentrations of many inflammatory molecules were higher in patients with AF, compared with controls, including IL-6, IL-8, TNF-α and MCP-1 [101] In a comparison with warfarin treatment, AF patients on rivaroxaban had decreased levels of inflammatory cytokines [109] In an observational study including patients with CKD stage 3b-4 newly diagnosed with AF, rivaroxaban treatment compared to Warfarin was associated with lower levels of serum markers of inflammation and also improved kidney function and stabilization/ regression of valve calcifications [110]
DVT/VTE	CRP, IL-6, IL-8, D-dimers, F1 + 2	VTE risk was associated with the levels of the inflammatory biomarkers CRP [102], IL-6 and IL-8 [103] Plasma concentration of D-dimer can be utilized as a diagnostic indicator of DVT [104]; however, low sensitivity has been reported in specific instances [105] Patients with high plasma levels of D-dimers were found to have a higher risk of VTE than patients with lower levels [106] In patients who had received rivaroxaban during total hip replacement surgery, urinary F1 + 2 levels at day 3 post-surgery were significantly higher in the group that experienced VTE than in the groups that did not, suggesting that urinary F1 + 2 levels could be used to stratify patients according to VTE risk [107]
HF	IL-8, TAT, D-dimers, F1 + 2	In patients with chronic systolic HF, a relationship was found between IL-8 levels and risks of adverse outcomes in patients [108] In patients with HF, levels of TAT, D-dimer and F1 + 2 have all been reported to increase over time; treatment of these patients with rivaroxaban was found to reduce the rate of increase of levels of D-dimer and TAT, and to reverse the increase in levels of F1 + 2 [19] In COMMANDER-HF, rivaroxaban reduced the risk of stroke in pts with D-dimer > 515 ng/mL [72]

*AF* atrial fibrillation; *CRP* C-reactive protein; *DVT* deep vein thrombosis; *F*1 + 2 prothrombin fragment 1 + 2; *HF* heart failure; *IL*-6 interleukin-6; *IL*-8 interleukin-8; *MCP*-1 monocyte chemoattractant protein-1; *TAT* thrombin–antithrombin complex; *TNF*-α, tumor necrosis factor-α; *VTE* venous thromboembolism

In addition to the molecular biomarkers of fibrin formation and coagulation, platelet activation, inflammation and endothelial changes discussed above, changes to concentrations of other molecular biomarkers have been found to be associated with specific disease states. As such, exploratory studies have investigated the potential effects of rivaroxaban exposure on levels of a variety of disease biomarkers.

Studies have suggested that AF is associated with systemic and cardiac oxidation and shares many of the same risk factors as atherosclerosis, a disease that is perpetuated by oxidative stress [111]. Preclinical studies have reported mixed results for the investigations of the effects of rivaroxaban on known markers of oxidative stress, such as malonaldehyde, reactive oxygen species, nitric oxide synthase isotype 2 and nitrogen oxide [87, 112, 113]. For example, in a rat model of peripheral-ischemia reperfusion, animals treated with rivaroxaban had significantly lower (*p* < 0.05) mean plasma levels of malondialdehyde (24.9 µmol/L) than sham mice (75.6 µmol/L). However, there were no significant differences between groups in plasma levels of nitrogen oxide [112].

An enhanced renin-angiotensin system causes hypertension, an important risk factor for chronic kidney disease. Treatment with rivaroxaban decreased the urinary albumin excretion and attenuated histologic changes of glomerular hypertrophy, mesangial matrix expansion, effacement of the podocyte foot process, and thickened glomerular basement membrane in hypertensive mice overexpressing renin. A renal protective effect of rivaroxaban provides an important clinical implication on the underlying mechanism by which rivaroxaban is associated with lower risks of decline in estimated glomerular filtration rate, doubling of serum creatinine, and acute kidney injury in patients with nonvalvular atrial fibrillation in clinical studies [114].

Finally, studies have investigated other biomarkers as a means of stratifying patients into groups that can gain the most benefit from rivaroxaban treatment. For example, in a post hoc analysis of the ATLAS ACS 2-TIMI 51 trial, patients with ACS were stratified by risk using biomarkers of high-risk disease [115]. Positive biomarker was predefined as either serum troponin concentration above the decision limit or serum creatine kinase–myocardial band isozyme above the upper limit, at normal level, or both. Based on the efficacy results, it was concluded that biomarker-positive patients with no previous history of stroke or transient ischemic attack may be an optimal target population to receive rivaroxaban in combination with antiplatelet therapy for secondary prevention of ACS. A recent study testing the efficacy of rivaroxaban versus aspirin for secondary stroke prevention in ESUS (Embolic Stroke of Undetermined Source) patients investigated whether hs-cTnT (high-sensitivity cardiac Troponin T) levels might be associated with major vascular events and if it may help to identify patients who would benefit from anticoagulation after ESUS (substudy of NAVIGATE-ESUS study). Here it was found that hs-cTnT is indeed associated with increased cardiovascular event rates which means that these biomarkers could support stratification of patients for cardiovascular risk, but not for decision -making regarding anticoagulant therapy [116]. Similarly, in the COMMANDER-HF trial, inclusion of patients in the study based on elevated plasma concentrations of natriuretic peptides (NPs) as selection criteria with the goal to support HF ascertainment and risk enrichment was performed. The results showed that elevated NPs for inclusion increased event rates allowing earlier completion of the trial but did not modify treatment effect with rivaroxaban [117]. In the VaLiDate-R study which started in January 2019 and is still ongoing at time of preparation of this manuscript, impaired endogenous fibrinolysis is assessed as potential novel biomarker for risk-stratification to identify patients who would benefit from more potent antithrombotic therapy [118].

### Outlook and novel approaches

In recent years, there has been an increase in the number of investigations using biomarkers to probe various biological processes, and future technical advances may contribute to further growth. For example, proteomic profiling techniques using mass spectrometry or affinity multiplexing assays could accelerate novel biomarker discovery. Proteomics studies can evaluate all proteins in a system, making them hypothesis-free and unbiased [119, 120]. A consensus statement on outcome parameters in AF trials has highlighted plasma proteomics as a potentially useful approach to identify novel drug effects in early phases of trials. This technique could be used to identify surrogates for understanding the pathophysiologic mechanisms underlying a given disease [121].

The potential of such proteomic studies is highlighted by results from recent investigations. For example, a study among participants from the Framingham Heart Study Offspring Cohort identified eight proteins associated with risk of incident AF after adjustment for age and sex. However, authors noted that further investigation would be required to confirm if any of these markers are mechanistically related to AF development [122]. In addition, a recent study tested a pragmatic biomarker discovery strategy that integrated automated clinical biobanking (using electronic health records) and proteomics. This study identified two potential biomarkers that robustly predicted HF across diverse clinical settings [123].

Further biomarker investigations are currently ongoing in samples of the COMPASS trial in which the efficacy of dual pathway inhibition with 2.5 mg twice daily rivaroxaban and aspirin in patients with coronary artery disease or peripheral arterial disease or both was demonstrated. Here, platelet aggregation, platelet activation and inflammation markers, thrombin generation kinetics and tissue factor-induced platelet–fibrin clot strength will be measured at baseline, and 4 and 12 weeks after randomization in order to evaluate if treatment with rivaroxaban is associated with a reduction in platelet activation and aggregation, inflammation and coagulation markers [124].

## Conclusion

Based on the studies identified and discussed in this review, there are several results that support the hypothesis that rivaroxaban has effects that extend beyond the coagulation pathway. In our review of preclinical and clinical studies, which tested the impact of rivaroxaban, we found several publications that reported results on biomarkers involved in various biological pathways and processes, such as biomarkers of coagulation status, including direct target engagement biomarkers (F1 + 2 and TAT complex) and indirect biomarkers (D-dimers).

In addition, and probably due to the interaction of the coagulation system with other biological pathways, our review identified various studies that have reported impacts of rivaroxaban on biomarkers of platelet effects, inflammation and endothelial changes. We found that the evidence for an effect of rivaroxaban on biomarkers of platelet activity, endothelial function, inflammation and oxidative stress derives predominantly from preclinical studies. Such results are useful for hypothesis generation but may not always match the human situation and consequently not translate into clinics. Therefore, additional animal studies and -most of all- clinical studies would be required.

Investigations of molecular biomarkers have limitations: There have been inconsistencies in the definitions used for different types of biomarkers. To address this problem, the FDA–National Institutes of Health working group developed the Biomarkers, EndpointS, and other Tools (BEST) resource in 2018, to provide guidance on the terminology for this type of research [8]. Another issue is the use of different types of assays for one analyte and of the results for inter-study comparisons – often with inconsistencies and lack of standardization between assays. A representative and well-investigated example are the high inter-laboratory and inter-method variabilities in D-dimer assays, likely due to the use of different reagents and the lack of standardized internationally certified calibrators and quality-control measures [125, 126]. Furthermore, depending on the state of the disease at the time of the blood draw, the results of the different biomarkers may vary which has to be considered and can limit their applicability for direct risk stratification in clinical practice. Translation to subsequent use as potential surrogate biomarkers requires further clinical validation investigations [8, 13].

In conclusion, the effect of rivaroxaban on D-dimers, TAT complex and F1 + 2 as well as inflammatory markers could be shown in clinical studies. Preclinical and clinical studies have also provided data, which showed effects of rivaroxaban on the pathways of platelet activation and endothelial changes. Future investigations will be required to further manifest these findings and potentially apply them in clinical situations.

## Data Availability

Not applicable.

## Abbreviations

ACS: Acute coronary syndrome
ADP: Adenosine diphosphate
AF: Atrial fibrillation
BEST: Biomarkers, EndpointS, and other Tools
DVT: Deep vein thrombosis
F1 + 2: Prothrombin fragment 1 + 2
FDA: US Food and Drug Administration
FU: Fibrinolytic units
FVIIa: Factor VIIa
FX: Factor X
FXa: Activated factor X
HF: Heart failure
hsIL-6: High sensitivity interleukin-6
ICAM-1: Intercellular adhesion molecule-1
IL-1β: Interleukin-1β
IL-6: Interleukin-6
LA/LAA: Left atrial/left atrial appendage
MCP-1: Monocyte chemoattractant protein-1
MMP: Matrix metalloproteinase
PAR: Protease-activated receptor
PCI: Percutaneous coronary intervention
PE: Pulmonary embolism
TAT: Thrombin–antithrombin
TF: Tissue factor
β-ThG: Thromboglobulin
TNF-α: Tumor necrosis factor-α
VCAM-1: Vascular cell adhesion molecule-1
vWF: Von Willebrand factor
